# Analysis of Immature (CD4^–^CD8^–^) Thymic Subsets in T-Cell Receptor *αß* Transgenic Mice

**DOI:** 10.1155/1992/45150

**Published:** 1992

**Authors:** Anne Wilson, Hanspeter Pircher, Pamela Ohashi, H. Robson Macdonald

**Affiliations:** 1 lnstitute of Pathology University Hospital Zurich 8091 Switzerland; 2 Ludwig Institute for Cancer Research Lausanne Branch Epalinges 1066 Switzerland

**Keywords:** T-cell development, T-cell receptor, transgenic mice, immature thymocytes

## Abstract

Introduction of a transgenic *αß* TCR (V*α*_2_, V*ß*_8.1_) specific for lymphocytic choriomeningitis virus (LCMV), in the context of H-2D^b^ into the genome of C57BL/6 mice, has many effects on the development and selection of T cells in both the thymus and the periphery. These mice produce increased numbers of CD4^–^8^+^ mature T cells, all of which express the transgenic TCR, and small numbers of CD4^–^8^+^cells using endogenous TCRs are also produced. This study follows the intrathymic development of T cells in these TCR *αß* transgenic mice, in particular the earliest CD4^–^8^–^ stages. As expected, the transgenic TCR is expressed on the cell surface at an earlier developmental stage than endogenous TCRs in nontransgenic littermate controls. Of the three major subsets expressing the heat–stable antigen (HSA), only the most mature, the CD25^–^CD44^–^ expresses the transgenic TCR, and the earlier CD25^–^CD44^+^ and CD25^+^CD44^–^ do not. Furthermore, in contrast to other TCR*αß*transgenic lines, TCR *γδ* lineage cells appear to develop normally.


*Developmental Immunology*, 1992, Vol. 2, pp. 85–94
Reprints available directly from the publisher
Photocopying permitted by license only

© 1992 Harwood Academic Publishers GmbH
Printed in the United Kingdom

### Analysis of Immature (CD4⁻CD8⁻) Thymic Subsets in T-Cell Receptor $\alpha\beta$ Transgenic Mice

ANNE WILSON*‡, HANSPETER PIRCHER†, PAMELA OHASHI†, and H. ROBSON MACDONALD‡

‡*Ludwig Institute for Cancer Research, Lausanne Branch, 1066 Epalinges, Switzerland*
†*Institute of Pathology, University Hospital, 8091 Zurich, Switzerland*

Introduction of a transgenic $\alpha\beta$ TCR ($V\alpha_2$, $V\beta_{8.1}$) specific for lymphocytic choriomeningitis virus (LCMV), in the context of H-2D$^b$ into the genome of C57BL/6 mice, has many effects on the development and selection of T cells in both the thymus and the periphery. These mice produce increased numbers of CD4$^-$8$^+$ mature T cells, all of which express the transgenic TCR, and small numbers of CD4$^+$8$^-$ cells using endogenous TCRs are also produced. This study follows the intrathymic development of T cells in these TCR $\alpha\beta$ transgenic mice, in particular the earliest CD4$^-$8$^-$ stages. As expected, the transgenic TCR is expressed on the cell surface at an earlier developmental stage than endogenous TCRs in nontransgenic littermate controls. Of the three major subsets expressing the heat-stable antigen (HSA), only the most mature, the CD25$^-$CD44$^-$ expresses the transgenic TCR, and the earlier CD25$^-$CD44$^+$ and CD25$^+$CD44$^-$ do not. Furthermore, in contrast to other TCR $\alpha\beta$ transgenic lines, TCR $\gamma\delta$ lineage cells appear to develop normally.

KEYWORDS: T-cell development, T-cell receptor, transgenic mice, immature thymocytes.

### INTRODUCTION

The development of mature T lymphocytes is a complex process, involving proliferation and differentiation of immature precursor cells through a series of quite well-defined stages. CD4$^-$CD8$^-$ prothymocytes (originating in the fetal liver or bone marrow) colonize the thymus and subsequently proliferate and differentiate to give rise to CD4$^+$8$^+$ intermediates and ultimately functional cells expressing either CD4 or CD8 (Fowlkes et al., 1985; MacDonald et al., 1988b; Scollay et al., 1988). During this process, the T-cell receptor (TCR) $\alpha\beta$ and $\gamma\delta$ genes are rearranged and expressed, and various other phenotypic markers are acquired or lost (Fowlkes and Pardoll, 1989; Pearse et al., 1989). Positive and negative selection events limit the repertoire of expressed TCR $\alpha\beta$ genes to avoid autoreactivity and account for MHC-restricted antigen recognition (Kappler and Marrack, 1987; von Boehmer, 1990). This latter selection phenomenon has come under closer scrutiny due to the recent availability of transgenic mice expressing TCR $\alpha\beta$ with defined antigen and MHC specificity (Sha et al., 1988; Teh et al., 1988; Berg et al., 1989; Kaye et al., 1989; Pircher et al., 1989a). In the present study, we have analyzed immature CD4$^-$CD8$^-$ T cell subsets that would normally not express surface TCR from TCR $\alpha\beta$ transgenic mice in order to assess the effects of premature TCR expression on early T-cell development.

*Corresponding author.

### RESULTS

### Increase of Immature (CD4$^-$8$^-$) Thymocytes in TCR Transgenic Mice

We have previously described transgenic mice expressing a TCR ($V_{\beta 8.1}$, $V_{\alpha 2}$) that is specific for LCMV in the context of H-2D$^b$ (Pircher et al., 1989a). On the selective MHC background (H-2$^b$), CD8$^+$ T cells bearing the transgene are positively selected (Pircher et al., 1989a; Ohashi et al., 1990). As shown in Fig. 1 and Table 1, such transgenic thymi are smaller than littermate controls in adult (8–12 wk) H-2$^b$ mice; however, the imma-

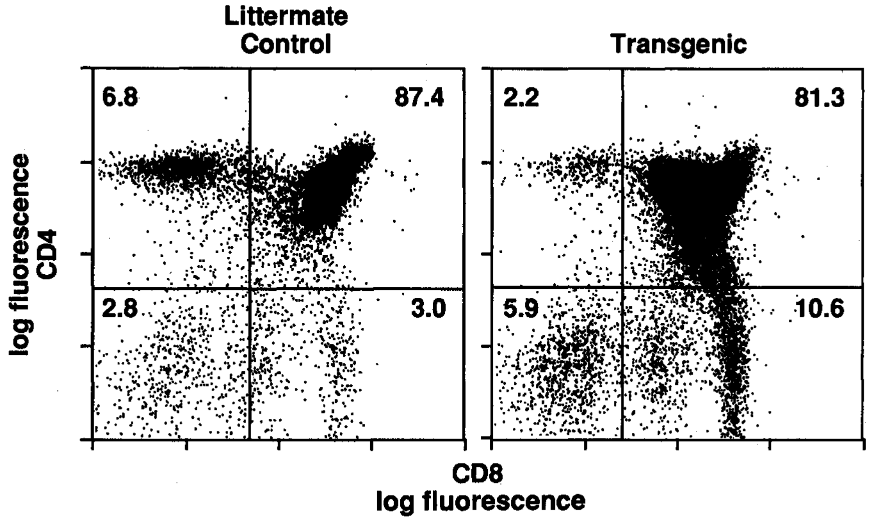

FIGURE 1. Comparison of the expression of CD4 and CD8 on adult thymocytes of H-2$^b$ TCR $\alpha\beta$ transgenic mice and their nontransgene-bearing littermate controls. Cells were stained with anti-CD4 PE and anti-CD8 FITC (both direct conjugates) and, after live gating by forward light scatter (FLS) and 90° side scatter (SSC) to exclude dead cells and debris, 25,000 cells were analyzed with the FACScan flow cytometer. Quadrant gates were set as indicated using the negative control (unstained) cells, and the numbers shown correspond to the percent of viable thymocytes in each quadrant.

ture CD4$^-$CD8$^-$ subset is increased both in percentage and in absolute numbers in the transgenics. On the H-2$^d$ background, where there is no positive selection of the transgene-bearing cells, the thymi are of a similar size to their littermate controls, but still have increased numbers and percentage of the CD4$^-$CD8$^-$ subset (Table 1).

### Expression of Transgenic TCR on CD4$^-$CD8$^-$ Thymocytes

The transgenic TCR expressed in these mice can be detected by mAbs directed against $V_{\beta 8.1}$ (KJ16) or $V_{\alpha 2}$ (B20.1). As shown in Fig. 2, a large proportion of CD4$^-$8$^-$ thymocytes expressed both the transgenic TCR $\alpha$ and $\beta$ chains. Furthermore, staining with mAbs directed against CD3 or TCR $\alpha\beta$ gave similar patterns as $V_{\beta 8.1}$ or $V_{\alpha 2}$ staining, indicating that few (if any) nontransgenic TCR $\alpha$ $\beta$ were expressed. The same staining patterns were observed on CD4$^-$8$^-$ thymocytes in the presence (H-2$^b$), or absence (H-2$^d$) of the selecting element, indicating that this has no effect on the expression of the transgenic TCR.

### Subsets of CD4$^-$8$^-$ Thymocytes in TCR Transgenic Mice

CD4$^-$8$^-$ thymocytes can be subdivided into subsets on the basis of expression of various surface markers. Among the most useful markers are heat–stable antigens (HSA), IL-2 receptor $\alpha$ chain (CD25), and Pgp-1 (CD44), which together define four major subsets (MacDonald et al., 1988b; Scollay et al., 1988). According to current views (Pearse et al., 1989; Petrie et al., 1990), HSA$^+$CD25$^-$CD44$^+$ immature precursors give rise sequentially to HSA$^+$CD25$^+$CD44$^-$ and subsequently HSA$^+$CD25$^-$CD44$^-$ subsets, the latter being immediate precursors of CD4$^+$8$^+$ cells (Wilson et al., 1989; Petrie et al., 1990). The fourth subset (HSA$^-$CD25$^-$CD44$^+$) is believed not to belong to the normal generative lineage and may result from an aberrant selection process (Crispe et al., 1987; Scollay et al., 1988).

TABLE 1

Thymus Subsets in TCR $\alpha\beta$ Transgenic Mice[a]

| Mice | Total thymus | CD4$^+$CD8$^-$ | CD4$^-$CD8$^+$ | CD4$^+$CD8$^+$ | CD4$^-$CD8$^-$ |
|---|---|---|---|---|---|
| H-2$^b$ Littermate | 170±28 | 12±3 | 7±3 | 144±27 | 4±1 |
| H-2$^b$ Transgenic | 92±23 | 4±1 | 13±4 | 71±10 | 7±2 |
| H-2$^d$ Littermate | 163±21 | 16±1 | 6±1 | 137±22 | 3±1 |
| H-2$^d$ Transgenic | 186±60 | 20±6 | 6±1 | 153±52 | 6±2 |

[a]Data expressed as absolute number of cells per thymus ($\times 10^{-6}$)±SD, and are the mean of four (H-2$^d$) and nine (H-2$^b$) experiments each using pools of two to eight mice.

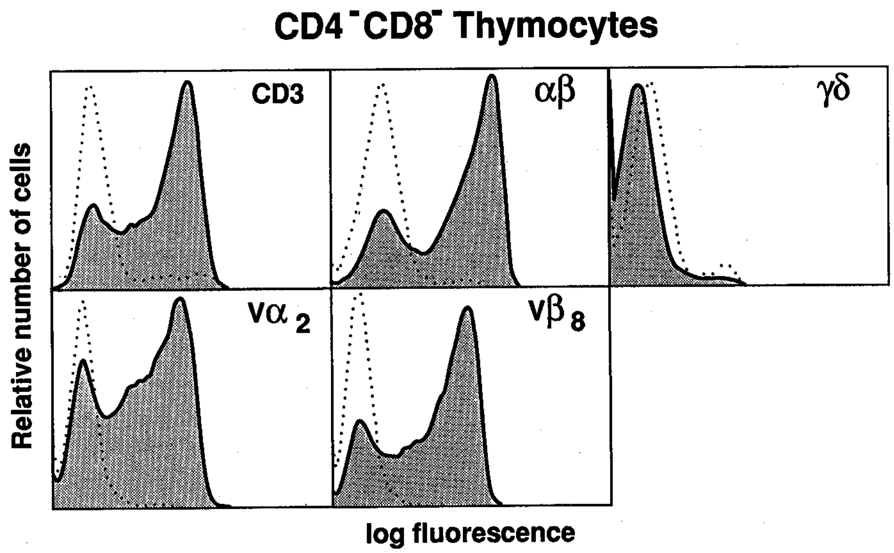

FIGURE 2. Expression of the TCR on immature ($CD4^-CD8^-$) thymocytes from H-$2^b$ $\alpha\beta$ transgenic mice and their littermate controls. Single-parameter profiles of surface TCR expression on purified $CD4^-CD8^-$ thymocytes were obtained by staining with culture supernatants followed by goat antirat FITC for the CD3 (17A2), $V\alpha_2$ (B20.1), and $V\beta_8$ (KJ16); and with biotin conjugates followed by PE-streptavidin for $\alpha\beta$ (H57) and $\gamma\delta$ (GL3). Solid lines correspond to transgenic and dotted lines to the littermate controls. Negative controls (second stages alone) have been omitted from this plot to simplify it, but were the same as the negative peaks for the littermate control.

Comparison of these major $CD4^-8^-$ subsets in adult H-$2^b$ TCR transgenic mice and littermate controls (Table 2, Fig. 3) revealed no significant differences in the two putatively most immature populations ($HSA^+CD25^-CD44^+$ and $HSA^+CD25^+CD44^-$). In contrast, the putatively most mature ($HSA^+CD25^-CD44^-$) $CD4^-8^-$ subset was substantially increased in absolute numbers in the transgenics, and the "aberrant" ($HSA^-CD25^-CD44^+$) subset was much reduced. Similar results were obtained from transgenic mice on the H-$2^d$ background.

### Expression of Transgenic TCR on $CD4^-8^-$ Subsets

The $CD4^-8^-$ subsets described before were analyzed for expression of the transgenic TCR. Transgenic TCR ($V\beta8.1$ or $V\alpha2$) was found on the relatively mature $HSA^+CD25^-CD44^-$ subpopulation and was not detected on the less mature $HSA^+CD25^-CD44^+$ and $HSA^+CD25^+CD44^-$ subsets (Fig. 4, and data not shown). The $HSA^-$ subset could not be analyzed for TCR expression in the transgenics due to the small number of cells present; however, this subset was readily detectable in the control littermates, where many cells expressed $V\beta8.2$ (data not shown; Budd et al., 1987; Fowlkes et al., 1987).

### Expression of TCR $\gamma\delta$ in TCR $\alpha\beta$ Transgenics

Several studies have suggested that expression of TCR $\beta$ transgenes leads to allelic exclusion of endogenous TCR $\gamma$ genes as well as TCR $\beta$ (Fenton et al., 1988; Uematsu et al., 1988; von Boehmer et al., 1988; Pircher et al., 1990). As shown in Fig. 5 and Table 3, TCR $\gamma\delta^+$ cells were readily detectable in the $CD4^-8^-$ subset of the H-$2^b$ or H-$2^d$ TCR $\alpha\beta$ transgenics described here. Although the relative percentage of TCR $\gamma\delta^+$ cells was lower in transgenics than in littermate controls, the absolute number was not significantly different between the two groups (Table

TABLE 2
Immature ($CD4^-CD8^-$) Thymus Subsets in TCR $\alpha\beta$ Transgenic Mice[a]

| Mice | $HSA^+CD25^-CD44^+$ | $HSA^+CD25^+CD44^-$ | $HSA^+CD25^-CD44^-$ | $HSA^-CD25^-CD44^+$ |
|---|---|---|---|---|
| H-$2^b$ Littermate | 0.19±0.03 | 2.59±0.65 | 1.27±0.11 | 0.43±0.24 |
| H-$2^b$ Transgenic | 0.21±0.05 | 2.87±0.92 | 3.89±0.07 | 0.17±0.08 |
| H-$2^d$ Littermate | 0.37* | 0.96±0.08 | 0.68±0.29 | 1.11±0.37 |
| H-$2^d$ Transgenic | 0.32* | 0.95±0.33 | 4.76±0.25 | 0.36±0.19 |

[a]Data expressed as absolute number of cells per thymus ($\times10^{-6}$), ±SD* only one value obtained. Results are the mean of three (H-$2^d$) and six (H-$2^b$) experiments each using pools of four to twelve mice.

3), thus suggesting that the TCR $\gamma\delta$ lineage develops normally in these TCR $\alpha\beta$ transgenic mice.

### Other Phenotypic Markers on TCR Transgenic CD4⁻8⁻ Thymocytes

We also compared the expression of other surface markers on H-$2^b$ TCR transgenic versus control $CD4^-8^-$ thymocytes (Fig. 6). Several markers that are normally associated with the degree of T-cell maturation (such as CD5 and Mel-14) were expressed at higher levels on the transgenic population. On the other hand, a marker associated with cell division (RL73) was expressed at reduced levels on transgenic versus control $CD4^-8^-$ cells. This latter result was confirmed by a reduced frequency of large blast cells (assessed by FLS) among $CD4^-8^-$ transgenic thymocytes (data not shown).

### Analysis of "Immature" CD4⁻8⁺ Thymocytes in TCR Transgenic Mice

In normal mice, differentiation of $CD4^-8^-$ to $CD4^+8^+$ thymocytes frequently occurs via a rapidly cycling $CD4^-8^+$ blast intermediate that expresses high levels of HSA and no detectable CD3 (MacDonald et al., 1988a; Shortman et al., 1988). As shown in Fig. 7, all $CD4^-8^+$ thymocytes in H-$2^b$ TCR transgenic mice expressed high levels of CD3 (as expected), and low levels of HSA as compared to control littermates. Furthermore, few blast cells were detectable (by FLS analysis) in the transgenic $CD4^-8^+$ population (Fig. 7). By these criteria, "immature" $CD4^-8^+$

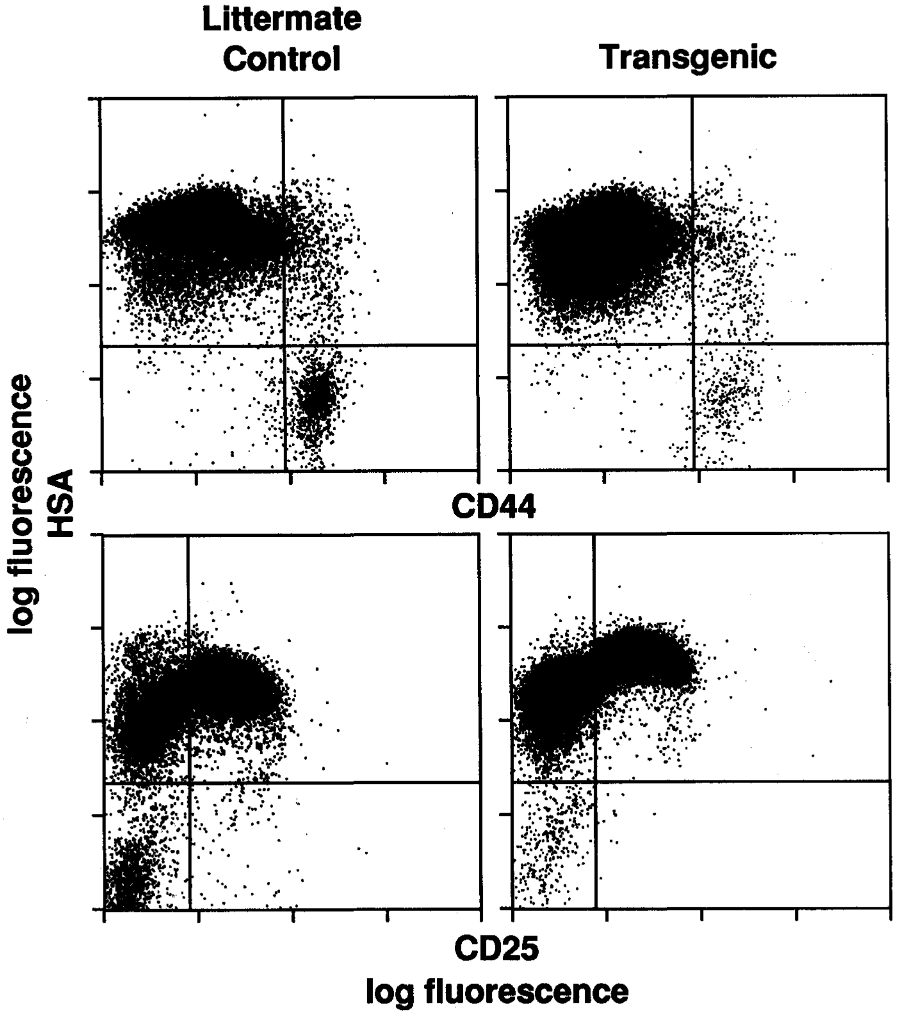

FIGURE 3. Immature thymus subsets defined by the surface expression of HSA, CD25, and CD44. $CD4^-CD8^-$ thymocytes from H-$2^b$ transgenic and littermate control mice stained in the top panel with anti-CD44 (PgP1) culture supernatant and goat antirat Ig FITC second stage, followed by rat Ig to block any free binding sites, then anti-HSA biotin conjugate followed by PE-streptavidin; and in the second panel with anti-HSA FITC and anti-CD25 PE, both direct conjugates. Live gating to exclude dead cells and debris was performed, quadrants were set using second stage alone or unstained cells respectively, and cells were analyzed as described for Fig. 1.

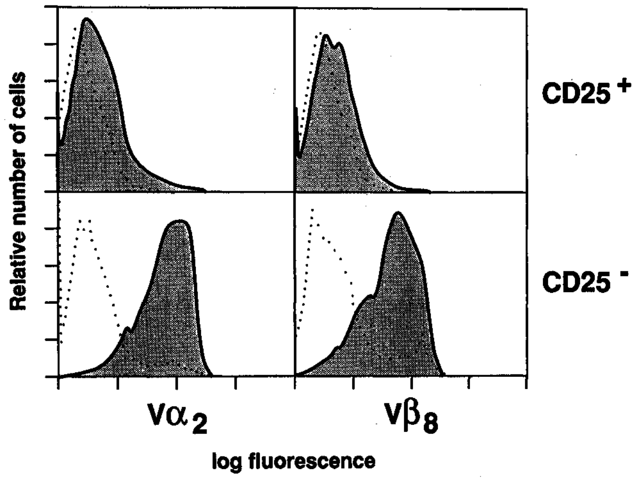

FIGURE 4. Expression of $V\alpha_2$ or $V\beta_8$ on subsets of H-2$^b$ CD4$^-$CD8$^-$ thymocytes. Purified CD4$^-$CD8$^-$ thymocytes were stained in three colors as follows: culture supernatants for the anti-TCR antibodies, second-stage fluorescein conjugates, and rat Ig for blocking were as described in Figs. 2 and 3; this was followed by anti-CD25-PE (direct conjugate), anti-HSA-biotin and finally TR-PE-avidin. Cells were analyzed and live gating performed as described in Fig. 1, except that 100,000 events were collected. The HSA$^+$CD25$^+$ and HSA$^+$CD25$^-$ were selected by a second gating and the TCR expression on these subpopulations obtained. Solid lines and shading correspond to transgenic and dotted lines to littermate controls.

thymocytes are lacking in the TCR transgenic thymus. However, CD4$^-$8$^+$ cells with a lower level of CD8 are found in the transgenic thymi in numbers comparable to that of the littermate controls (1.5±0.4% and 1.6±0.6%, respectively; see Fig. 1). As noted elsewhere, this phenotype may correspond to relative immaturity of these cells (Shortman et al., 1988). The recently described CD4$^+$8$^-$3$^-$ "immature" thymocyte showing a similar phenotype and morphology (Shortman et al., 1988; Matsumoto et al., 1989; Hugo et al., 1990) was also virtually undetectable in these transgenic mice.

### DISCUSSION

It is well established that the precursors of mature T lymphocytes are contained within the CD4$^-$8$^-$ thymocyte subset (Fowlkes et al., 1985; Crispe et al., 1987; Shimonkevitz et al., 1987; Scollay et al., 1988). The developmental sequence occurring within that population is now more clearly understood (Pearse et al., 1989; Petrie et al., 1990), although the precise events that direct this differentiation are not. In this communication, we have analyzed CD4$^-$8$^-$ thymocytes from mice expressing a transgenic TCR $\alpha\beta$ in order to gain further insights into early T-cell development. In normal mice, TCR $\alpha\beta$ is first expressed at the CD4$^+$8$^+$ stage of thymus development and hence TCR expression in transgenic mice allows cross-correlations with other phenotypic markers believed to define early developmental stages.

The current consensus view of lineage relationships among major CD4$^-$8$^-$ subsets (Fowlkes and Pardoll, 1989; Petrie et al., 1990) holds that HSA$^+$CD25$^-$CD44$^+$ early precursors differentiate through a HSA$^+$CD25$^+$CD44$^-$ intermediate stage to become HSA$^+$CD25$^-$CD44$^-$ blast cells prior to acquisition of CD4 and/or CD8. Consistent with this model, the present results indicate that only the latter (presumably most mature) CD4$^-$8$^-$ population expresses detectable transgenic TCR, and the putatively more immature CD4$^-$8$^-$ subsets do not. In contrast to our findings, Teh et al. (1990) have recently reported expression of transgenic TCR $\alpha\beta$ on essentially all CD4$^-$8$^-$ thymocytes. The reason for this apparent discrepancy is not clear; however, it should be noted that the TCR $\alpha$ and TCR $\beta$ transgenic constructs used by Teh et al. (1990) were large genomic fragments (Teh et al., 1988; Uematsu et al., 1988), whereas the TCR constructs used here were TCR $\alpha$ and $\beta$ cDNA clones coupled to a H-2K promoter and Ig heavy-chain enhancer (Pircher et al., 1989b). It is thus possible that these differences in regulatory elements lead to differential ontogeny of expression of the two TCR transgenes in vivo. Failure to express the transgene on the early CD4$^-$8$^-$ subsets is not due to lack of function of the H-2K promoter because H-2K$^b$ is expressed at high levels on all of these subsets (data not shown).

In contrast to the three major HSA$^+$ subsets of CD4$^-$8$^-$ thymocytes, HSA$^-$CD4$^-$8$^-$ cells appear not to belong to the generative lineage (Scollay et al., 1988; Fowlkes and Pardoll, 1989). Furthermore, this HSA$^-$ subset is peculiar in that it appears late in development and most of the cells express TCR $\alpha\beta$ with a strong preference for the $V_{\beta 8.2}$ variable domain (Budd et al., 1987; Fowlkes et al., 1987). Interestingly, HSA$^-$CD4$^-$8$^-$ thymocytes were dramatically reduced in our TCR $\alpha\beta$ transgenic mice. In contrast, Teh et al. (1990) reported large numbers of HSA$^-$ thymocytes (~20% of

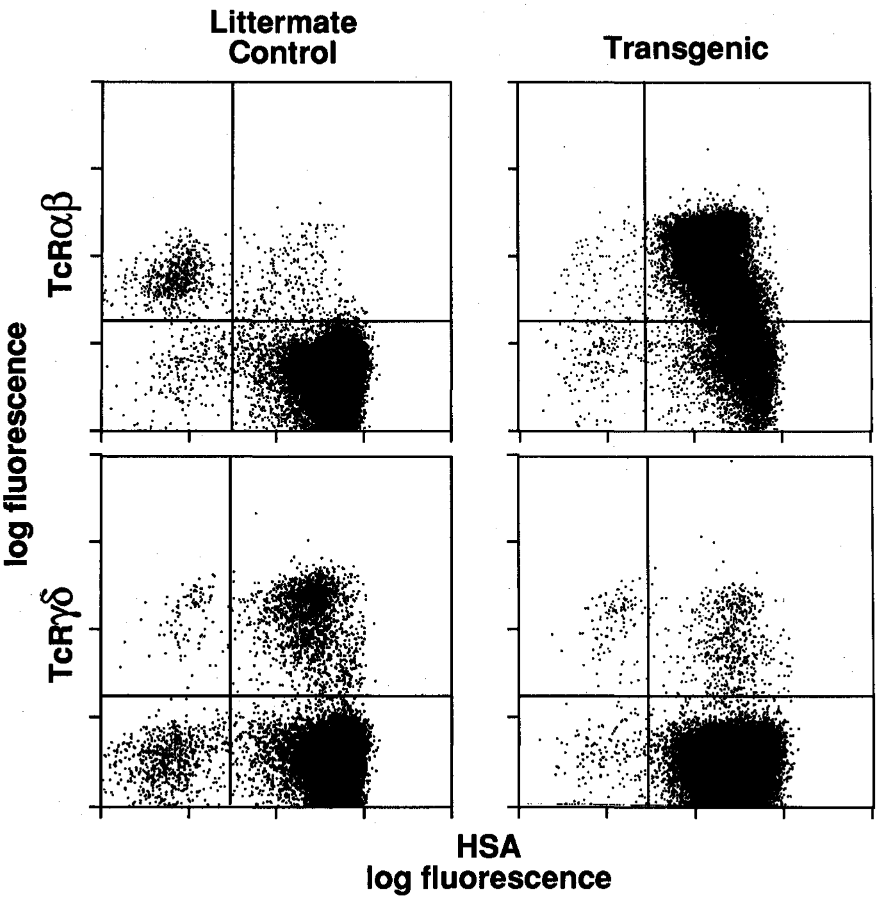

FIGURE 5. Prescence of TCR $\gamma\delta^+$ cells in the H-2[b] CD4⁻CD8⁻ thymus population. Expression of TCR $\alpha\beta$ or TCR $\gamma\delta$ correlated with HSA expression on CD4⁻CD8⁻ thymocytes from transgenic or littermate controls. Cells were stained with anti-HSA FITC (direct conjugate) and anti-TCR $\alpha\beta$ or anti-TCR $\gamma\delta$ biotin conjugates, followed by PE-Av. Live gating to exclude dead cells and debris, quadrants to show the position of the negative controls, and analyses were performed as described in Fig. 1.

total adult $CD4^-8^-$ cells) in their TCR $\alpha\beta$ transgenic mice. In interpreting this dramatic difference, it is noteworthy that the transgenic TCR $\beta$ chain of Teh et al. (1990) encodes $V_{\beta 8.2}$, whereas the $\beta$ chain studied here encodes $V_{\beta 8.1}$. Thus, it may be that $V_{\beta 8.2}$ expression is essential (even in transgenic mice) for the development of the $HSA^-CD4^-8^-$ subset. Whether this subset arises via aberrant positive selection of $V_{\beta 8.2}{}^+CD4^+CD8^+$ thymocytes (von Boehmer, 1990) or by some other mechanism remains to be determined.

TABLE 3
TCR Expression by $CD4^-CD8^-$ Thymocytes from TCR $\alpha\beta$ Transgenic Mice[a]

| Mice | $CD3^+$ | TCR $\alpha\beta^+$ | TCR $\gamma\delta^+$ |
|---|---|---|---|
| H-2[b] Littermate | 6.4±0.9 | 2.5±0.2 | 3.8±0.6 |
| H-2[b] Transgenic | 57.4±1.1 | 55.1±3.3 | 2.8±0.8 |
| H-2[d] Littermate | 12.1±3.0 | 11.8±1.8 | 2.4±0.3 |
| H-2[d] Transgenic | 52.1±4.3 | 54.3±0.5 | 2.2±0.8 |

[a]Data expressed as absolute number of cells per thymus ($\times 10^{-5}$) ±SD, and are the mean of four experiments, each using pools of four to eight mice.

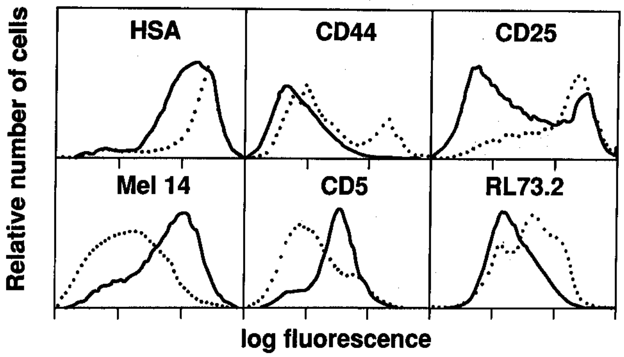

FIGURE 6. Changes in the level of expression of other phenotypic markers. Single-parameter profiles of various surface antigens on total CD4⁻CD8⁻ thymocytes from the H-2[b] transgenics or their littermate controls. Cells were stained with culture supernatants, followed by a goat antirat Ig FITC conjugate. Shaded profiles are from transgenics and the unshaded from the littermate controls.

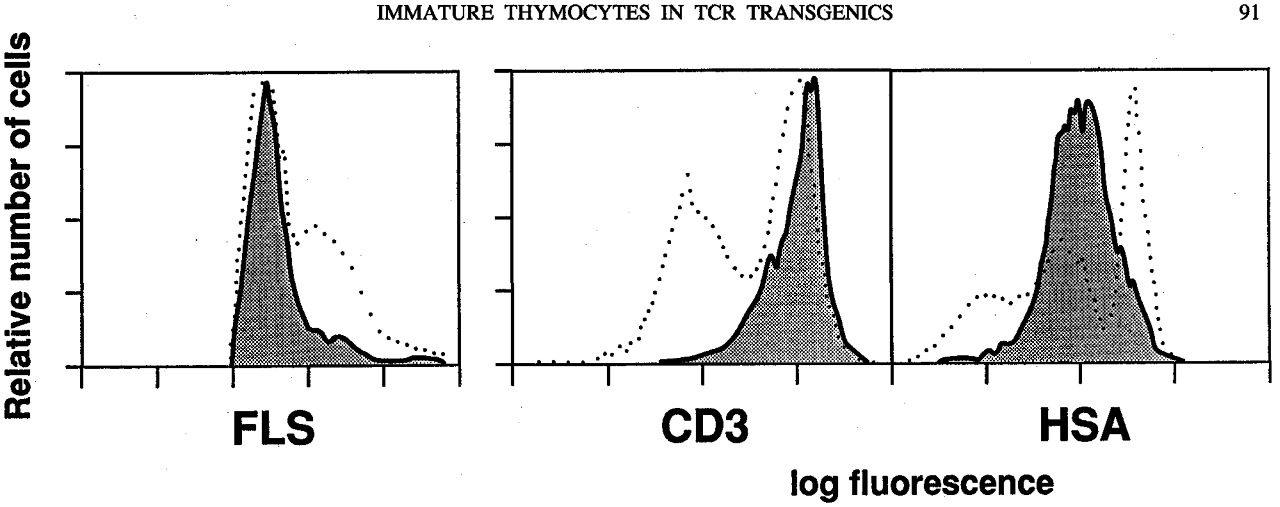

FIGURE 7. The "immature" CD4⁻CD8⁺ thymocyte. CD4⁻ thymocytes were isolated from H-2$^b$ transgenics or their littermate controls and stained in three colors with, in the following order, anti-CD3 (17A2 culture supernatant), goat antirat Ig PE, rat Ig for blocking, anti-HSA FITC, anti-CD8 biotin, and finally TR-PE-Av. Live gating to exclude dead cells and debris was performed as in Fig. 1, then the CD8$^+$ cells were selected by a second live gate on the FL3$^+$ (TR-PE-Av$^+$) cells. Single-parameter profiles are shown for the FLS (reflecting relative cell size), CD3, and HSA expression of the CD4$^-$CD8$^+$ thymocytes. Solid lines correspond to transgenic and dotted lines to littermate controls.

Transition of thymocytes from the CD4$^-$8$^-$ to CD4$^+$8$^+$ compartment may involve an intermediate CD4$^-$8$^+$ (MacDonald et al., 1988a; Shortman et al., 1988) or CD4$^+$8$^-$ (Hugo et al., 1990; Matsumoto et al., 1989) cell expressing little or no detectable surface TCR. In mouse, the "immature CD8$^{+}$" population is rapidly cycling and exhibits high levels of HSA. By these criteria, no immature CD4$^-$8$^+$ thymocytes could be detected in TCR $\alpha\beta$ transgenic mice. The absence of this intermediate subset may result from accelerated differentiation of the immediate CD4$^-$8$^-$ precursors of these cells. Indeed, HSA$^+$CD25$^-$CD44$^-$ thymocytes were present in elevated numbers in TCR $\alpha\beta$ transgenic mice of both the H-2$^b$ (selecting) and H-2$^d$ (nonselecting) backgrounds. In addition, this subset expressed enhanced levels of several phenotypic markers (CD5, Mel-14) and a decreased proportion of cycling cells, both traits that are usually associated with T-cell maturation. Therefore, the premature expression of surface transgenic TCR, even if it cannot be positively selected (as on the H-2$^d$ background), may be associated with accelerated maturation of immature thymocytes. Furthermore, these data provide evidence that the "immature," CD4$^+$8$^-$3$^-$ or CD4$^-$8$^+$3$^-$ subsets are not obligatory stages in the differentiation from CD4$^-$8$^-$ to CD4$^+$8$^+$ cells (Wilson et al., 1989; Petrie et al., 1990).

Considerable controversy surrounds the developmental relationship between TCR $\alpha\beta$ and TCR $\gamma\delta$ lineages (Fowlkes and Pardoll, 1989). In this context, several studies have indicated that introduction of a rearranged TCR $\beta$ transgene effectively inhibits further gene rearrangement at the TCR $\gamma$ locus, presumably due to some transacting allelic exclusion mechanism (Fenton et al., 1988; von Boehmer et al., 1988), and other reports have suggested that a partial inhibitory effect of transgenic TCR $\beta$ on TCR $\gamma$ rearrangement occurs, but that the TCR $\gamma\delta$ population remains unchanged (Pircher et al., 1990). In other studies using $\gamma$ and/or $\gamma\delta$ transgenic mice, complete inhibition (Bonneville et al., 1989), no effect (Dent et al., 1990; Ishida et al., 1990), or enhancement (Ferrick et al., 1989) of the TCR $\gamma\delta$ lineage have been reported. In the TCR $\alpha\beta$ transgenic mice reported here, TCR$\gamma\delta^+$ cells were readily detected in the CD4$^-$8$^-$ thymocyte subset and absolute numbers of these cells were identical to the values obtained in control littermates. In addition, the TCR $\gamma\delta$-expressing dendritic epidermal T cells (DETC) isolated from the skin of these TCR $\alpha\beta$ transgenic mice were shown to be indistinguishable (both in absolute numbers and V$\gamma_3$ usage) from those found in their littermate controls (P. Ohashi et al., unpublished data). Thus, it seems that TCR $\alpha\beta$ and TCR $\gamma\delta$ lineages are controlled independently in these animals; however, it is possible that TCR $\gamma\delta$ cells develop normally due to a delayed expression of the TCR $\alpha\beta$ transgene (see before).

In conclusion, the premature expression of a defined TCR $\alpha\beta$ transgene provides a useful model system in which to test the predicted developmental lineage of immature (CD4$^-$8$^-$) thymocyte subsets. Furthermore, the comparison of CD4$^-$8$^-$ thymocytes expressing different transgenic V$\beta$ domains may ultimately lead to new insights regarding the developmental origin of the HSA$^-$CD4$^-$8$^-$ subset.

### MATERIALS AND METHODS

### Mice

All the mice used in this study were bred and maintained at the Swiss Institute for Experimental Cancer Research (Epalinges, Switzerland). The transgenic TCR (V$\alpha_2$,J$\alpha_{TA31}$/V$\beta_{8.1}$,D$\beta$,J$\beta_{2.4}$, C$\beta_2$) was derived from a LCMV/H-2D$^b$-specific cytotoxic T-cell clone P14 (Pircher et al., 1987). Transgenic males on either a C57BL/6 (H-2$^b$) or Balb/c (H-2$^d$) genetic background (Pircher et al., 1989a) were heterozygously bred with normal MHC compatible 6–8-week-old females. In this way, a colony of TCR $\alpha\beta$ transgenic mice on the selective (H-2$^b$) or nonselective (H-2$^d$) background and their nontransgene bearing littermates were established. Litters were screened for transgene-bearing mice at approximately 4 weeks of age by staining peripheral blood lymphocytes with the monoclonal antibody KJ16, which binds the V$\beta_{8.1}$ and V$\beta_{8.2}$ TCRs, followed by goat antirat Ig-FITC, and then counterstaining with a mixture of anti-CD8 biotin and either anti-CD4-PE or anti-CD4 biotin and finally PE-streptavidin. The percent of V$\beta_8$-bearing T cells was 15–20% for the nontransgene-bearing littermates and 85–90% for the transgenics.

### Isolation of Immature Thymocytes

CD4$^-$CD8$^-$ thymocytes were purified from total thymus by treating cell suspensions at 5×10$^7$/mL in DMEM supplemented with glutamine and Hepes buffer and containing 1% FCS, with previously determined concentrations of the anti-CD4 mAb RL1.172.4 (Ceredig et al., 1985) and the anti-CD8 mAb 3.168.8.1 (Sarmiento et al., 1982) for 20 min on ice, followed by 45 min at 37 °C after the addition of rabbit complement (final concentration 1/20, supplied by The Buxted Rabbit Company, Buxted, UK) and 10 units/mL of DNase I (Boehringer Mannheim GmbH, Mannheim, West Germany) to prevent excessive clumping of dead cells and debris. Dead cells were removed by centrifugation over Isopaque-Ficoll (Pharmacia, Uppsala, Sweden). To ensure purity of the final preparation, this entire procedure was repeated with the cells at 10$^7$/mL. CD4$^-$ thymocytes were prepared in the same manner with the omission of the anti-CD8 antibody. In both cases, the resultant cells were greater than 98% pure.

### Monoclonal Antibodies

Culture supernatants were prepared from hybridomas grown in the laboratory as follows. Anti-CD3, 17A2 (Miescher et al., 1989/1990); anti-V$\alpha_2$, B20.1 (B. Malissen et al., manuscript in preparation); anti-V-$\beta_{8.1,8.2}$, KJ16.133.18 (Haskins et al., 1984); anti-CD44 (PgP1), IM7.8.1 (Trowbridge et al., 1982); anti-CD25 (IL2R$\alpha$), PC61.5 (Ceredig et al., 1985); Mel-14 (Gallatin et al., 1983); anti-CD5, 53-7.3 (Ledbetter and Herzenberg, 1979); and RLI.73.2 (MacDonald et al., 1985). Biotin conjugates were prepared in the laboratory, and were as follows: anti-CD8, 53.6.72 (Ledbetter and Herzenberg, 1979); anti-CD4, GK1.5 (Dialynas et al., 1983); anti-HSA, M1/69 (Springer et al., 1978); and anti-CD25 (as before). Anti-TCR $\alpha\beta$, H57.597 (Kubo et al., 1989) and anti-TCR $\gamma\delta$, GL3 (Goodman and Lefrancois, 1989), both biotin conjugates, were the kind gift of J.-P. Rosat, WHO Immunology Research and Training Unit, Epalinges, Switzerland. Fluorescein (FITC) conjugates of anti-CD8, H35-17.2 (Goldstein et al., 1982) and anti-HSA, M1/69 (as before) were prepared in the laboratory. Anti-CD4-PE was purchased from Bectin Dickinson, Mountain View, CA. Second-stage reagents were purchased as follows: Goat antirat Ig-FITC (Tago, Inc., Burlingame, CA), Goat antirat Ig-PE (Southern Biotechnology Associates, Inc., Birmingham, AL), PE-Av (R-phycoerythrin-streptavidin, from Caltag Laboratories, San Francisco, CA), and PE-TR-Avidin (R-phycoerythrin (PE), covalently bound to texas red (TR), and coupled to avidin; with an excitation wavelength characteristic of PE and emission wavelength characteristic of TR; from Southern Biotechnology Associates, Birmingham, AL).

### Staining Procedures

Single two-color and three-color staining of thymocytes was performed as previously described (Shortman et al., 1988; Wilson et al., 1988, 1989). Analysis of these stained cells was done with a FACS II for the single staining and a FACScan for the two- and three-color fluorescence (both Flow Cytometers were supplied by Becton Dickinson, Mountain View, CA).

### ACKNOWLEDGMENTS

We thank Christian Knabenhans and Pierre Zaech for their patience and skill with the flow cytometric analysis, Bernard Malissen for his kind gift of the B20.1 monoclonal antibody, and Anna Zoppi for her help in preparation of the manuscript.

*(Received May 14, 1991)*

*(Accepted September 25, 1991)*

### REFERENCES

Berg L.J., Frank G.D., and Davis M.M. (1990). The effects of MHC gene dosage and allelic variation on T cell receptor selection. Cell **60:** 1043–1046.

Bonneville M., Ishida I., Mombaerts P., Katsuki M., Verbeek S., Berns A., and Tonegawa S. (1989). Blockage of $\alpha\beta$ T-cell development by TCR $\gamma\delta$ transgenes. Nature **342:** 931–934.

Budd R.C., Miescher G.C., Howe R.C., Lees R.K., Bron C., and MacDonald H.R. (1987). Developmentally regulated expression of T cell receptor $\beta$ chain variable domains in immature thymocytes. J. Exp. Med. **166:** 577–582.

Ceredig R., Lowenthal J.W., Nabholz M., and MacDonald H.R. (1985). Expression of interleukin-2 receptors as a differentiation marker on intra-thymic stem cells. Nature **314:** 98–100.

Crispe I.N., Moore M.W., Husmann L.A., Smith L., Bevan M.J., and Shimonkevitz R.P. (1987). Differentiation potential of subsets of $CD4^-CD8^-$ thymocytes. Nature **329:** 336–339.

Dent A.L., Matis L.A., Hooshmand F., Widacki S.M., Bluestone J.A., and Hedrick S.M. (1990). Self-reactive $\gamma\delta$ T cells are eliminated in the thymus. Nature **343:** 714–719.

Dialynas D.P., Wilde D.B., Marrack P., Pierres A., Wall K.A., Havran W., Otten G., Loken M.R., Pierres M., Kappler J., and Fitch F.W. (1983). Characterisation of the murine antigenic determinant, designated L3T4a, recognised by monoclonal antibody GK1.5: Expression of L3T4a by functional T cell clones appears to correlate primarily with class II MHC antigen reactivity. Immunol. Rev. **74:** 29–56.

Fenton R.G., Marrack P., Kappler J.W., Kanagawa O., and Seidman J.G. (1988). Isotypic exclusion of $\gamma\delta$ T cell receptors in transgenic mice bearing a rearranged $\beta$-chain gene. Science **241:** 1089–1092.

Ferrick D.A., Suryaprakash R.S., Balhausen W., Iwamoto A., Pircher H., Walker C.L., Yokoyama W.M., Miller R.G., and Tak T.W. (1989). T cell function and expression are dramatically altered in T cell receptor $V\gamma_{1.1}J\gamma_4C\gamma_4$ transgenic mice. Cell **57:** 483–492.

Fowlkes B.J., and Pardoll D.M. (1989). Molecular and cellular events of T cell development. Adv. Immunol. **44:** 207–264.

Fowlkes B.J., Edison L., Mathieson B.J., and Chused T.M. (1985). Early T lymphocytes: Differentiation in vivo of adult intrathymic precursor cells. J. Exp. Med. **162:** 802–822.

Fowlkes B.J., Kruisbeek A.M., Ton-That H., Weston M.A., Coligan J.E., Schwartz R.H., and Pardoll D.M. (1987). A novel population of T-cell receptor $\alpha\beta$ bearing thymocytes which predominantly express a single $V\beta$ gene family. Nature **329:** 251–254.

Gallatin W.M., Weissman I.L., and Butcher E.C. (1983). A cell surface molecule involved in organ-specific homing of lymphocytes. Nature **304:** 30–34.

Goldstein P., Goridis C., Schmitt-Verhulst A.-M., Hayot B., Pierres A., van Agthoven A., Kaufmann Y., Eshhar Z., and Pierres M. (1982). Lymphoid cell surface interaction structures detected using cytolysis-inhibiting monoclonal antibodies. Immunol. Rev. **68:** 5–42.

Goodman T., and Lefrancois L. (1989). Intraepithelial lymphocytes. Anatomical site, not T cell receptor form, dictates phenotype and function. J. Exp. Med. **170:** 1569–1581.

Haskins K., Hannum C., White J., Roehm N., Kubo R., Kappler J., and Marrack P. (1984). The antigen-specific, major histocompatibility complex-restricted receptor on T cells. J. Exp. Med. **160:** 452–471.

Hugo P., Waanders G.A., Scollay R., Shortman K., and Boyd R.L. (1990). Ontogeny of a novel $CD4^+CD8^-CD3^-$ thymocyte subpopulation: A comparison with $CD4^-CD8^+CD3^-$ thymocytes. Int. Immunol. **2:** 209–218.

Ishida I., Verbeek S., Bonneville M., Itohara S., Berns A., and Tonegawa S. (1990). T-cell receptor $\gamma\delta$ and $\gamma$ transgenic mice suggest a role of a $\gamma$ silencer in the generation of $\alpha\beta$ T cells. Proc. Natl. Acad. Sci. USA **87:** 3067–3071.

Kappler J.W., and Marrack P. (1987). The T cell receptor. Science **238:** 1073–1079.

Kaye J., Hsu M.-L., Sauron M.-E., Jameson S.C., Gascoigne N.R., and Hedrick S.M. (1989). Selective development of $CD4^+$ T cells in transgenic mice expressing a class II MHC-restricted antigen receptor. Nature **341:** 746–749.

Kubo R., Born W., Kappler J.W., Marrack P., and Pigeon M. (1989). Characterisation of a monoclonal antibody which detects all murine $\alpha\beta$ T cell receptors. J. Immunol. **142:** 2736–2742.

Ledbetter J.A., and Herzenberg L.A. (1979). Xenogeneic monoclonal antibodies to mouse lymphoid differentiation antigens. Immunol. Rev. **47:** 63–90.

MacDonald H.R., Budd R.C., and Howe R.C. (1988a). A $CD3^-$ subset of $CD4^-CD8^+$ thymocytes: A rapidly cycling intermediate in the generation of $CD4^+CD8^+$ cells. Eur J. Immunol. **18:** 519–523.

MacDonald H.R., Howe R.C., Pedrazzini T., Lees R.K., Budd R.C., Schneider R., Liao N.-S., Zinkernagel R.M., Louis J.A., Raulet D.K., Hengartner H., and Meischer G. (1988b). T-cell lineages, repertoire selection and tolerance induction. Immunol. Rev. **104:** 157–182.

MacDonald H.R., Lees R.K., and Bron C. (1985). Cell surface glycoproteins involved in the stimulation of interleukin-1 dependent interleukin-2 production by a subline of EL4 thymoma cells. I. Functional characterization by monoclonal antibodies. J. Immunol. **135:** 3944–3950.

Matsumoto K., Yoshikai Y., Matsuzaki G., Asano T., and Nomoto K. (1989). A novel $CD3^-J11d^+$ subset of $CD4^+CD8^-$ cells repopulating thymus in radiation bone marrow chimeras. Eur. J. Immunol. **19:** 1203–1207.

Miescher G.C., Schreyer M., and MacDonald H.R. (1989/1990). Production and characterization of a rat monoclonal antibody against the murine CD3 molecular complex. Immunol. Lett. **23:** 113–118.

Ohashi P.S., Pircher H., Bürki K., Zinkernagel R.M., and Hengartner H. (1990). Distinct sequence of negative or positive selection implied by thymocyte T-cell receptor densities. Nature **346:** 861–863.

Pearse M., Wu L., Egerton M., Wilson A., Shortman K., and Scollay R. (1989). A murine early thymocyte developmental sequence is marked by transient expression of the interleukin 2 receptor. Proc. Natl. Acad. Sci. USA **86:** 1614–1618.

Petrie H.T., Hugo P., Scollay R., and Shortman K. (1990). Lineage relationships and developmental kinetics of immature thymocytes: CD3, CD4, and CD8 acquisition in vivo and in vitro. J. Exp. Med. **172:** 1583–1588.

Pircher H., Baenziger J., Schilham M., Sado T., Kamisaku H., Hengartner H., and Zinkernagel R.M. (1987). Characterization of virus-specific cytotoxic T cell clones from allogeneic bone marrow chimeras. Eur. J. Immunol. **17:** 159–166.

Pircher H., Bürki K., Lang R., Hengartner H., and Zinkernagel R.M. (1989a). Tolerance induction in double specific T-cell receptor transgenic mice varies with antigen. Nature **342:** 559–561.

Pircher H., Mak T.W., Lang R., Balhausen W., Ruedi E., Hengartner H., Zinkernagel R.M., and Bürki K. (1989b). T cell tolerance to Mls$^a$ encoded antigens in T cell receptor V$\beta$ $_{8.1}$ chain transgenic mice. EMBO J. **8:** 719–727.

Pircher H., Ohashi P., Meischer G., Lang R., Zikopoulos A., Bürki K., Mak T.W., McDonald H.R., and Hengartner H. (1990). T cell receptor (TcR) $\beta$ chain transgenic mice: Studies on allelic exclusion and on the TcR+ $\gamma/\delta$ population. Eur J. Immunol. **20:** 417–424.

Sarmiento M., Dialynas D.P., Lancki D.W., Wall K.A., Lorber M.I., Loken M.R., and Fitch F.W. (1982). Cloned T lymphocytes and monoclonal antibodies as probes for cell surface molecules active in T cell-mediated cytolysis. Immunol. Rev. **68:** 135–170.

Scollay R., Wilson A., D'Amico A., Kelly K., Egerton M., Wu L., and Shortman K. (1988). Developmental status and reconstitution potential of subpopulations of murine thymocytes. Immunol. Rev. **104:** 81–120.

Sha W.C., Nelson C.A., Newberry R.D., Kranz D.M., Russell J.H., and Loh D.Y. (1988). Selective expression of an antigen receptor on CD8-bearing T lymphocytes in transgenic mice. Nature **335:** 271–274.

Shimonkevitz R.P., Husmann L.A., Bevan M.J., and Crispe I.N. (1987). Transient expression of IL-2 receptor precedes the differentiation of immature thymocytes. Nature **329:** 157–159.

Shortman K., Wilson A., Egerton M., Pearse M., and Scollay R. (1988). Immature CD4$^-$CD8$^+$ murine thymocytes. Cell. Immunol. **113:** 462–479.

Springer T., Galfre G., Secher D.S., and Milstein C. (1978). Monoclonal xenogeneic antibodies to murine cell surface antigens: Identification of novel leukocyte differentiation antigens. Eur. J. Immunol. **8:** 539–551.

Teh H.S., Kishi H., Scott B., Borgulya P., von Boehmer H., and Kisielow P. (1990). Early deletion and late positive selection of T cells expressing a male specific receptor in T-cell receptor transgenic mice. Dev. Immunol. **1:** 1–10.

Teh H.S., Kisielow P., Scott B., Kishi H., Uematsu Y., Blüthmann H., and von Boehmer H. (1988). Thymic major histocompatibility complex antigens and the $\alpha\beta$ T-cell receptor determine the CD4/CD8 phenotype of T cells. Nature **335:** 229–233.

Trowbridge I., Lesley J., Schulte R., Hyman R., and Trotter J. (1982). Biochemical characterisation and cellular distribution of a polymorphic, murine cell surface glycoprotein expressed on lymphoid tissues. Immunogenetics **15:** 299–312.

Uematsu Y., Ryser S., Dembic Z., Borgulya P., Krimpenfort P., Berns A., von Boehmer H., and Steinmetz M. (1988). In transgenic mice the introduced functional T cell receptor B gene prevents expression of endogenous $\beta$ genes. Cell **52:** 831–841.

von Boehmer H. (1990). Developmental biology of T cells in T cell-receptor transgenic mice. Annu. Rev. Immunol. **8:** 531–536.

von Boehmer H., Bonneville M., Ishida I., Ryser S., Lincoln G., Smith R.T., Kishi H., Scott B., Kisielow P., and Tonegawa S. (1988). Early expression of a T-cell receptor $\beta$-chain transgene suppresses rearrangement of the V$\gamma_4$ gene segment. Proc. Natl. Acad. Sci. USA **85:** 9729–9732.

Wilson A., D'Amico A., Ewing T., Scollay R., and Shortman K. (1988). Subpopulations of early thymocytes: A cross correlation flow-cytometric analysis of adult mouse Ly-2$^-$L3T4$^-$ (CD4$^-$CD8$^-$) thymocytes using eight different surface markers. J. Immunol. **140:** 1461–1469.

Wilson A., Petrie H.T., Scollay R., and Shortman K. (1989). The acquisition of CD4 and CD8 during the differentiation of early thymocytes in short-term culture. Int. Immunol. **1:** 605–612.

